# Design and Evaluation of Two-Stage Membrane-Separation Processes for Propylene–Propane Mixtures

**DOI:** 10.3390/membranes12020163

**Published:** 2022-01-29

**Authors:** Takehiro Yamaki, Nguyen Thuy, Nobuo Hara, Satoshi Taniguchi, Sho Kataoka

**Affiliations:** Research Institute for Chemical Process Technology, National Institute of Advanced Industrial Science and Technology (AIST), AIST Tsukuba Central 5, 1-1-1 Higashi, Tsukuba 305-8565, Ibaraki, Japan; nguyen.thuy@aist.go.jp (N.T.); n-hara@aist.go.jp (N.H.); taniguchi-satoshi@aist.go.jp (S.T.)

**Keywords:** membrane separation, process design, CO_2_ emissions, total annual cost, propylene

## Abstract

Propylene is industrially produced in a mixture with propane and generally separated from the mixture via distillation. However, because distillation is an energy-consuming process, a more efficient separation process should be developed to mitigate both carbon dioxide (CO_2_) emissions and production costs. In this study, a two-stage membrane-separation process was designed, and its CO_2_ emission and production costs were evaluated. The separation processes were designed to minimize energy consumption using different membrane combinations (two recently developed membranes each). To evaluate the separation processes using various membrane combinations, two indicators, i.e., CO_2_ emissions and total annual costs (TACs), were estimated based on the process simulation (Pro/II, version 10.1.1) results, including energy consumptions, operation expenditure, and capital expenditure. These results were compared to the distillation processes as benchmarks, and the advantages of the membrane-separation process were discussed. In the comparison, carbon taxes were implemented for assessing these two independent indicators as a single indicator, i.e., TAC with carbon tax. Furthermore, using the same scheme, model membranes were also employed in the two-stage membrane-separation process as case studies of technological forecasts.

## 1. Introduction

Propylene is one of the most important feed stocks in the chemical industry. It can be industrially produced via various reactions such as naphtha pyrolysis, methanol to olefine (MTO), and propane dehydrogenation [1,2,3,4,5,6]. In these reactions, propylene is obtained in a mixture containing propane and other byproducts. High-purity propylene is then obtained by separating it from the mixture. Distillation is commonly used to separate propylene from the mixture. However, it is one of the most energy-consuming processes in the propylene production, resulting in large carbon dioxide (CO_2_) emissions in the production processes. Thus, several alternative separation processes have been examined, including heat-pump assisted distillation [7,8,9,10,11], adsorptive separation [12,13,14,15,16], and membrane separation [17,18,19,20]. Among them, membrane separation is one of the most promising separation methods for propylene–propane mixtures [21]. In previous studies [17,18,19,20], membrane separation was compared to the conventional distillation process (CDiC) and vapor-recompression column (VRC). The results showed that, to replace conventional methods, further improvement of the membrane is still required for the following reasons [17,22,23]: (1) In a membrane-separation system, there is a trade-off relationship between the purity and recovery ratio of the products. When the recovery ratio of a product is small, high-purity product is obtained and vice versa because membrane separation is a unit operation based on the difference in the permeance of each component through the membrane. At a small permeate flow rate (i.e., low recovery ratio), only the component with a high permeance is obtained (i.e., high-purity product). Conversely, at a large permeate flow rate (i.e., high recovery ratio), components with a low permeance are obtained in the permeate stream (i.e., low purity). To obtain a high recovery ratio of highly pure product, membrane separation is commonly combined with other unit operations (i.e., hybrid processes), and/or multi-stage membrane-separation processes are used. Previous studies demonstrated that the energy consumption of hybrid processes consisting of membrane separation and distillation is significantly smaller than that of the single distillation process [24,25,26,27,28]. In our previous study [29], the energy consumption of multi-stage membrane-separation process was compared to a hybrid process with a feed stream containing propylene with different concentrations (30, 60, 90, and 98 mol%). The results showed that the two-stage membrane-separation process has a better performance compared to the hybrid process, especially when the propylene concentration was 90 mol% in the feed stream. Similarly, the present study focuses on two-stage membrane processes. (2) Multiple factors, such as separation factor and permeance, are required for the design of two-stage membrane-separation processes. According to the Robeson plot [30], membranes with a higher separation factor tend to have a small permeance. This trade-off relationship intricately influences the design of the two-stage membrane-separation processes. The separation factor and permeance affect the energy consumption during the membrane-separation process and the total area of the membrane, respectively. In our previous study [29], a two-stage membrane-separation process was designed using membranes with high separation factors, mainly to minimize the energy consumption. The process design using membranes with different separation performances needs to be considered.

The goal for designing the membrane separation process is to simultaneously fulfill the requirement of product purity and recovery ratio while reducing the energy consumption and the required membrane area. Furthermore, to simultaneously examine the energy consumption and membrane area, practical indicators other than energy consumption should be considered. Because the energy consumption and membrane area are closely related to the production cost, the total annual costs (TACs) were introduced as an indicator. In addition, CO_2_ emissions related to the energy consumption in the process should be examined to assess the environmental impact of the process.

In this study, a two-stage membrane-separation process that combines membranes with different separation factors and permeances was designed and evaluated based on its CO_2_ emissions and TACs. The membranes were selected for the separation process based on their performance values reported in the literature. These values were also used in each evaluation. Furthermore, model membranes were postulated as case studies for forecasting this separation process. The optimal combination of each membrane with the lowest TAC that includes the carbon tax (hereafter referred to as TAC with carbon tax) was identified for each membrane. Finally, the advantages of the membrane-separation process compared to the distillation processes were discussed.

## 2. Materials and Methods

### 2.1. Problem Statement

Propylene is separated by a two-stage membrane-separation process from a propylene–propane mixture obtained by a MTO reaction to have polymer-grade propylene (purity = 99.5 mol%). The recovery ratio of the propylene is 99.5%. The CO_2_ emissions and TACs of the two-stage membrane-separation processes are assessed compared to CDiC and VRC as benchmarks.

### 2.2. Membranes

**Figure 1:**
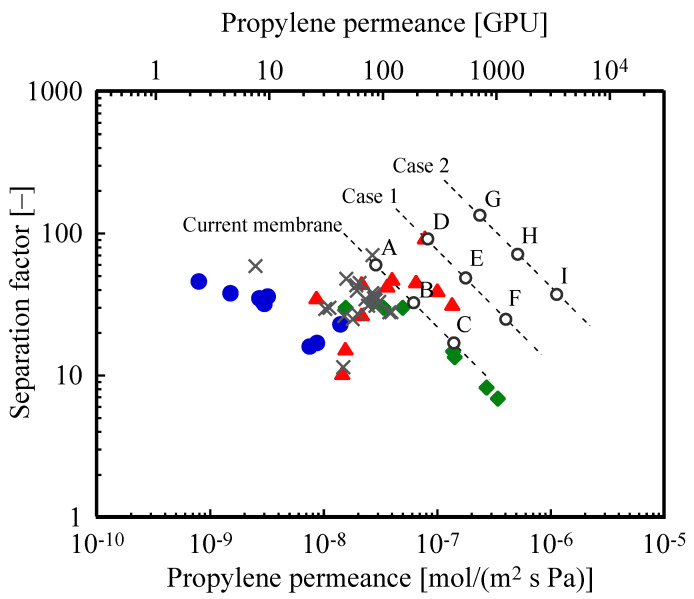
The relationship between propylene permeance and separation factor. O: membranes used in this study.

Various membrane types have been proposed for the separation of propylene–propane mixture, including carbon [31,32,33], silica [34], zeolite [35], and metal organic framework (MOF) [36,37,38,39,40,41] membranes. All these membrane types are selective of propylene over propane. Figure 1 shows the literature values of the propylene permeance and separation factor. The membranes of which the permeabilities have been reported without information on their thicknesses were not plotted in the figure because the permeability alone cannot be converted to permeance.

This figure is also known as the Robeson plot and is commonly used in studies of the relationship between the permeance and selectivity of membranes [30]. For example, silica membranes (green) have a relatively high propylene permeance with a low separation factor compared to MOF membranes (MOF is represented by gray crosses in Figure 1). The permeance and separation factor affect the membrane area and energy consumption, respectively. Simply, a separation process using a membrane with high permeance requires a small area of a membrane. A separation process using a membrane with a high separation factor consumes a small amount of energy. Membranes with high permeance and separation factors are desirable. However, it is difficult to develop a membrane with both merits. In this study, the propylene permeance and separation factor of the recently developed membranes indicated with the dashed line in the Figure 1 are employed for the design and evaluation (hereafter referred to as “current membrane”). Moreover, the membrane separation performances were assumed to be constant with respect to the operation pressure. In this membrane category, three levels of separation performance were defined: (A) high separation factor with low permeance; (B) intermediate separation factor and permeance; and (C) low separation factor with high permeance. In addition to the above current membrane, two hypothetical categories of separation performances from emerging innovation (Cases 1 and 2) were investigated. The details of Cases 1 and 2 are described in Section 3.2. Note that the combinations are limited to the same membrane category (e.g., A and B; E and F); those between different membrane categories (e.g., A and D; B and H) were not investigated. The separation performances of the membranes used in the calculation are summarized in Figure 1 and Table 1.

### 2.3. Membrane-Separation Process

To ensure that the membrane meets the requirements of the system, a single-stage membrane-separation process was examined before the design of the main membrane-separation process. In our previous study [29], a single-stage membrane-separation process was found to meet the product specifications (propylene purity 99.5 mol%, propylene recovery ratio 99.5%) at a high propylene concentration (98 mol%) in the feed, which was obtained from naphtha pyrolysis. Moreover, it was confirmed that the single-stage membrane-separation process could not simultaneously offer product purity and high recovery ratio at a 90 mol% propylene concentration in the feed. Thus, a two-stage membrane-separation process (hereafter referred to as the membrane-separation process) is used in this study.

**Figure 2:**
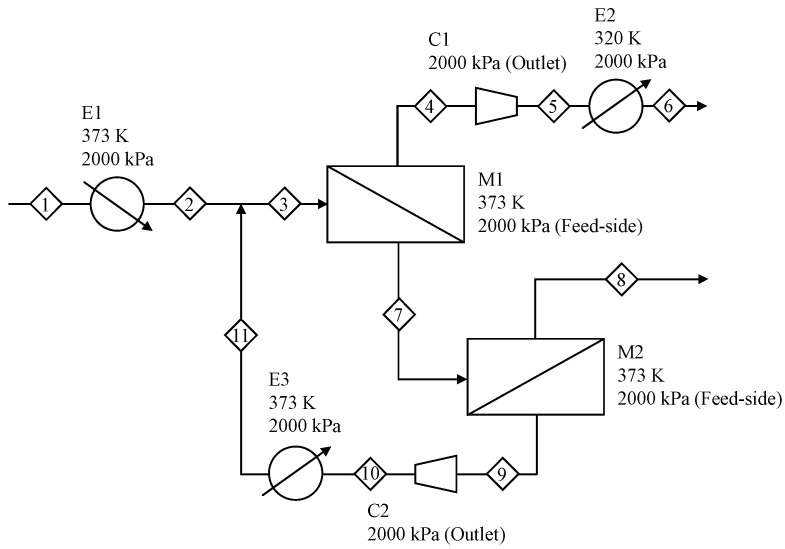
Schematic of the membrane-separation process. E: heat exchanger, M: membrane unit, C: compressor.

Figure 2 shows a schematic of the membrane-separation process [20]. The propylene–propane mixture is heated using a heater (E1) and fed to the first membrane unit (M1). Because the pressure on the permeate side of the first membrane unit is lower than that on the feed side, the permeate stream (Stream 4) is compressed using a compressor (C1) and then cooled using a cooler (E2) to adjust the pressure of the product requirement. Propylene is recovered from Stream 6, and the retentate stream (Stream 7) is fed to the second membrane unit (M2). Propane is recovered from Stream 8, and the permeate stream (Stream 9) is compressed using a compressor (C2), cooled using the cooler (E3), and then refed to the first membrane unit (M1). Note that the membrane-separation process, where membrane A is used in M1, and membrane B is used in M2, is denoted AB. The parameters of the membrane-separation process are listed in Table 2.

The material balance and energy balance of the processes, including membrane separators, compressors, and heat exchangers, were conducted using a process simulator (Pro/II, version 10.1.1). The simulation model for membrane separation was also used in Pro/II. The Peng–Robinson model was used to estimate the physical properties of propylene and propane because the membrane-separation process was operated at high pressure. The binary parameters of the Peng–Robinson model were loaded to the databank in Pro/II.

### 2.4. Design and Evaluation Scheme

**Figure 3:**
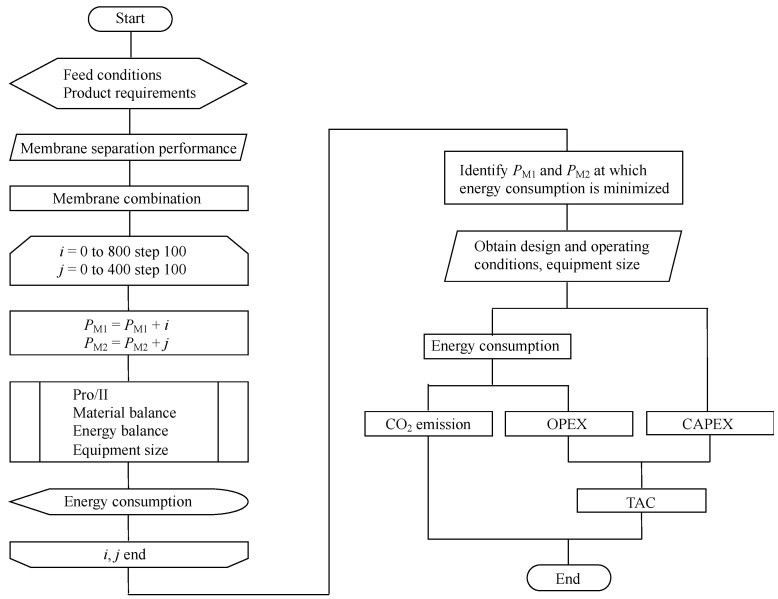
Design and evaluation scheme of the membrane-separation process.

Figure 3 shows the design and evaluation scheme of the separation process. First, the feed conditions and product requirements were selected (Table 2). The membrane combinations were constructed based on the membrane-separation performances (Table 1). A process simulation was conducted using Pro/II to determine the permeate-side pressure of M1 and M2 at the lowest energy consumption. The initial value of the permeate-side pressure of M1 (*P*_M1_) and M2 (*P*_M2_) was 200 kPa, which were increased 9 and 5 times, respectively, with a step size of 100 kPa. Thus, 45 times calculations were conducted. Next, the design and operating conditions were obtained at *P*_M1_ and *P*_M2_ when the energy consumption was minimized. The CO_2_ emissions were calculated based on the energy consumption. The operation expenditure (OPEX) was calculated based on the energy consumption; the capital expenditure (CAPEX) was calculated based on the design conditions and equipment size. Subsequently, the areas of the M1 and M2 membranes were simply identified using *P*_M1_ and *P*_M2_ when the fixed feed conditions and the product requirements were set as the membrane-separation performances. TAC was calculated based on OPEX and CAPEX, and TAC with carbon tax was calculated based on the carbon price and TAC.

### 2.5. Distillation Benchmark

CDiC and VRC were designed as benchmarks and used for comparison. The parameters of CDiC are listed in Table 3. The design parameters (numbers of stages and a feed stage) of VRC are the same as those of CDiC. The reflux and compression ratios were 16.10 and 1.4, respectively. The condenser and compressor duties were 34 GJ/h and 4169 kW, respectively.

### 2.6. Evaluation of CO_2_ Emissions and TAC

The CO_2_ emissions were estimated based on the energy consumption. Utilities were set as follows: medium pressure steam (reboiler, heater), refrigerated water (condenser, cooler), and electricity (compressor). The CO_2_ emission factors were adopted from the Inventory Database for Environmental Analysis (IDEA) [42] (Table 4).

TAC is typically calculated as follows (Equation (1)):

TAC = OPEX + CAPEX/Payback period (1)

OPEX and CAPEX were defined in this study as the utility cost and equipment purchase price, respectively. OPEX and CAPEX were calculated based on Turton’s method [43]. Here, only utility costs were considered as OPEX. The utility costs are also listed in Table 4. The equipment size required to evaluate CAPEX was calculated using the process simulator. The price of the membrane includes the cost of the membrane module. The OPEX and CAPEX were calculated based on the following assumptions:

- The payback (depreciation) period = 4 y.
- The annual operating time = 8000 h.
- The compressor is a single stage one.
- The compression efficiency = 0.75.
- The overall heat transfer coefficient = 0.671 kW/(m^2^ K) [44].
- The price of current membrane = 500 $/m^2^ [20].
- The price of the Case 1 membrane = 1000 $/m^2^.
- The price of the Case 2 membrane = 2000 $/m^2^.

## 3. Simulation Results and Discussion

### 3.1. Current Membranes

**Figure A1:**
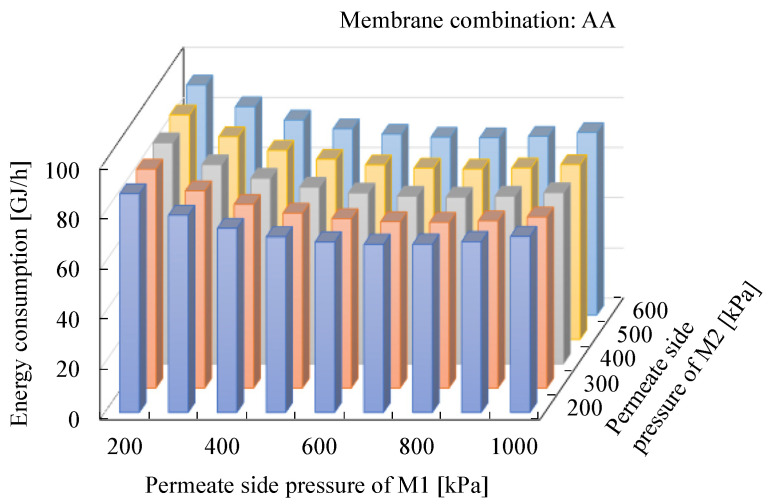
Energy consumption of AA at different permeate-side pressures of M1 and M2.

The driving force of the membrane-separation process is the partial pressure difference of the permeate components in the feed side and permeate side. The permeate flow rate (*F*_p_) is generally expressed as follows:

(2) $$F_{p}y_{p}=P_{i}A\left(p_{h}x_{r}-p_{l}y_{p}\right)$$

where *y*_p_, *P*_i_, *A*, *p*_h_, *x*_r_, *p*_l_, and *y*_p_ are the mole fraction of the component in the permeate stream, permeance of the component, membrane area, pressure at the feed side, mole fraction of the component in the retentate stream, pressure at the permeate side, and mole fraction of the component in the permeate stream, respectively. In this study, the components are propylene and propane. While a low and high pressure at the permeate and feed sides, respectively, are desirable for maintaining a large pressure difference, this leads to an increase in the compressor duty. Thus, the optimal permeate-side pressure must be determined to minimize energy consumption. As an example, the energy-consumption calculations of AA for different PM1 and PM2 are shown in Appendix A (Figure A1). The same calculations were performed for all membrane combinations. The design and operating conditions calculated at the lowest energy consumption for each membrane combination are summarized in Appendix B (Table A1). The values in Appendix B were used for the rest of the evaluations in this study.

**Figure 4:**
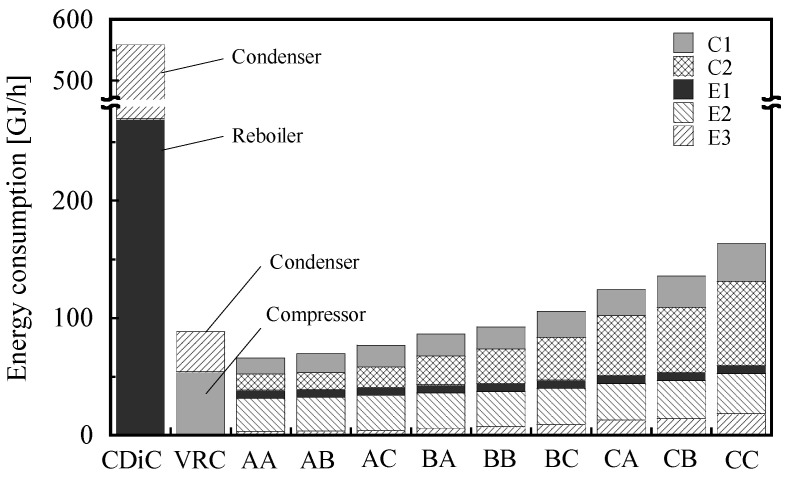
Energy consumptions of the two-stage membrane process with various combinations. C1 and C2 are compressors. The compressor duties are converted to primary energy. E1 is a heater; E2 and E3 are coolers. The energy consumption of CDiC includes the condenser and reboiler duties. The energy consumption of VRC includes the condenser and compressor duties.

Figure 4 shows the energy consumption for all membrane combinations. The membrane combinations with a high separation factor, such as AA and AB, exhibited low energy consumption (especially in C1, C2, and E3). This can be attributed to the higher permeate-side pressure and the reduced recycle flow rate (stream 9). The energy consumption of CDiC was significantly higher than that of the membrane-separation process. However, the energy consumption of VRC was considerably lower than that of CDiC and comparable to that of the membrane-separation process. The energy consumptions of AA, AB, AC, and BA were slightly lower (2 to 25% lower) than those of VRC. Because the energy consumption of CDiC was exceptionally large, only VRC was employed hereafter as a benchmark for the comparison to the membrane-separation processes.

**Figure 5:**
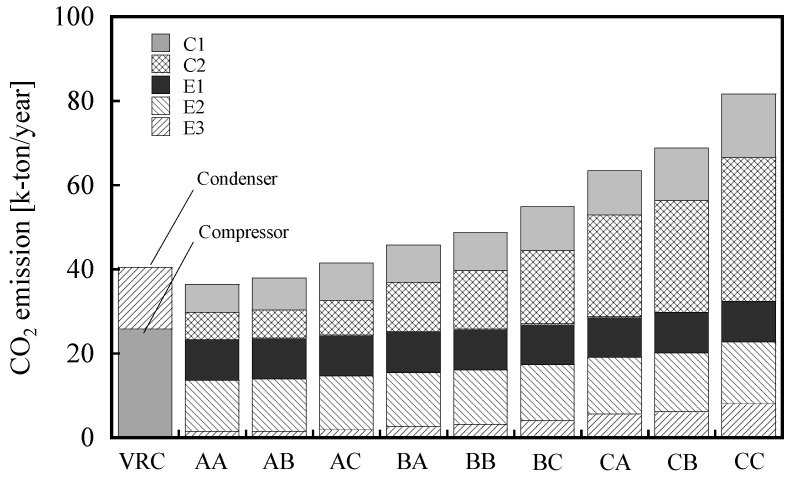
CO_2_ emissions from the two-stage membrane process with various combinations. C1 and C2 are the compression of the permeate stream using electricity. E1 is the feed heating using steam. E2 and E3 represent the cooling of the compressed permeate streams using refrigerated water.

Figure 5 shows the CO_2_ emissions for each membrane combination. The CO_2_ emissions of all membrane cases exhibit the same trend as that of the energy consumption shown in Figure 4. The CO_2_ emissions in the membrane systems are mainly caused by electricity, steam, and refrigerated water, which are the major components responsible for energy consumption. The CO_2_ emissions of AA and AB are 6 to 10% lower than those of VRC. The CO_2_ emissions of AC and BA are higher than those of VRC, although their energy consumptions are lower than those of VRC, because steam, electricity, and refrigerated water have different CO_2_-emission factors (Table 4).

**Figure 6:**
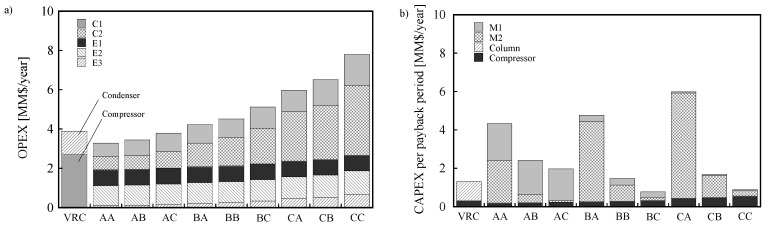
The production cost of the two-stage membrane process with various combinations: (**a**) OPEX and (**b**) CAPEX per payback period. The payback period = 4 y.

Figure 6 shows the calculated values of OPEX and CAPEX per payback period. OPEX also showed similar trend to that of the energy consumption (Figure 4) and CO_2_ emissions (Figure 5) because both OPEX and CO_2_ emissions are closely related to energy consumption. Low OPEX was observed for the membrane combinations with a high separation factor, such as AA and AB. Conversely, a completely different trend was observed for CAPEX compared to the CO_2_ emissions and OPEX. The CAPEX of AA and AB were higher than those of VRC. AA and AB have membranes with low permeance, which require large membrane areas that result in high CAPEX. The prices of the heat exchangers (E1, E2, and E3) were negligible, and hence, were not included in the graph. BC and CC used membranes with high permeance, which require small membrane areas, and result in low capital costs for M1 and M2. This trend is caused by the trade-off relationship between the separation factor and the permeance of the membrane, as described in the introduction. In other words, when the membrane with a high separation factor is used, the energy consumption is small, and consequently, OPEX is also small. On the other hand, when the membrane with high permeance is used, the required membrane area becomes small, resulting in less CAPEX.

**Figure 7:**
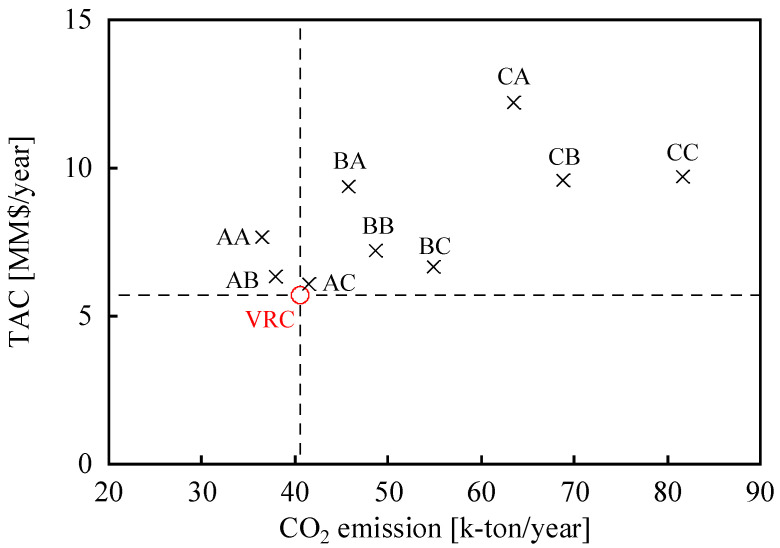
The relationship between CO_2_ emissions and TACs. O: VRC, ×: membrane-separation process using the current membranes.

Because OPEX and CAPEX show different trends, TAC, which was calculated using Equation (1), was plotted against CO_2_ emissions (Figure 7) for further evaluations. When the membrane with a high separation factor was used for M1 (e.g., AA, AB, and AC), the TAC was small. Similarly, when the membrane with a high permeance was used for M2 (e.g., AC, BC, and CC), the TAC was small. This can be attributed to the contribution of both OPEX and CAPEX to TAC. More importantly, the separation performances of the preferred membrane for M1 and M2 are different in the membrane separation process to reduce TAC. Overall, AC exhibited the lowest TAC among all cases of the membrane-separation processes. However, even the TAC of AC was still higher than that of VRC. Although the TAC of AA and AB were higher than that of VRC, the CO_2_ emissions of AA and AB were smaller than those of VRC.

From an environmental point of view, AA and AB, of which the CO_2_ emissions were smaller, may be desirable even if their TACs are higher than those of VRC. A small amount of CO_2_ emissions can be an advantage in terms of production cost specially when the carbon tax is implemented. To further evaluate this, a factor comprising TAC with the carbon tax, which is expected to be universally implemented in the near future, was estimated based on the CO_2_ emissions and carbon price. Various organizations established recommendations based on the carbon price; for example, for 2050, the International Monetary Fund has suggested a carbon price between 40 and 150 $/t-CO_2_ (depending on the country) [45]. In this study, a carbon price range of 0 to 200 $/t-CO_2_ was used.

**Figure 8:**
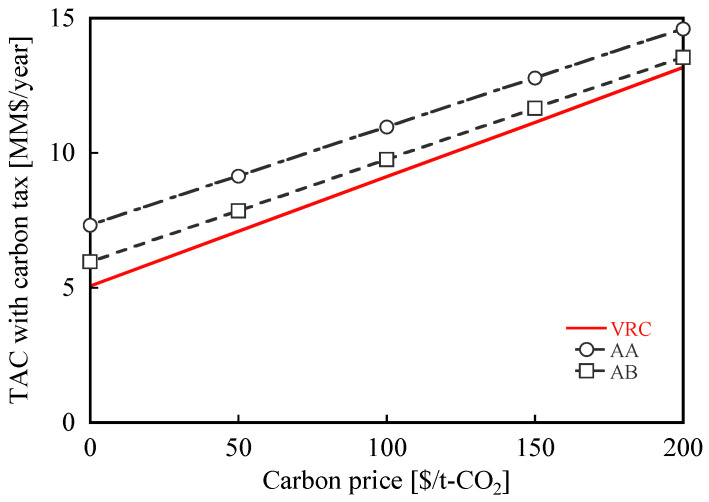
TACs with the carbon tax for different cases of the membrane-separation process compared to VRC.

Figure 8 shows TAC with the carbon tax plotted against the carbon price. TAC with the carbon tax of VRC was still lower than that of AA and AB over the entire range up to 200 $/t-CO_2_. TACs with the carbon tax of AA and AB become smaller than that of VRC when the carbon price was greater than 549 and 351 $/t-CO_2_, respectively, which is an unrealistic assumption according to several recommendations. Thus, even when the carbon price is considered, the membrane-separation process using the current membranes is less competitive than VRC regarding both CO_2_ emissions and TACs with carbon tax. Based on these results, the current membranes still need improvements to be able to replace VRC.

### 3.2. Case Studies of Technological Forecasts

As mentioned in Section 1, there is a trade-off relationship between the separation factor and permeance of membranes. Improving both parameters by technological innovation reduces both the CO_2_ emissions and TAC of the membrane-separation process compared to those of the current membrane. As case studies of technological forecasts, two membrane categories (namely, Cases 1 and 2) were examined (Table 1 and Figure 1). The membrane-separation processes employing Cases 1 and 2 were designed and evaluated in the same scheme of the investigation using the current membranes.

**Figure 9:**
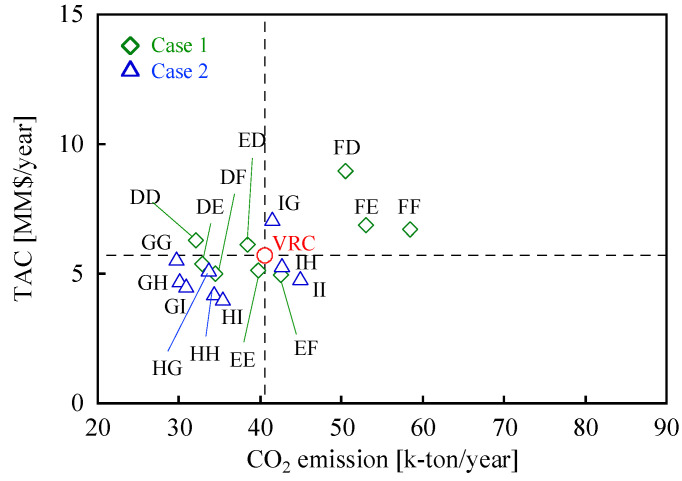
The relationship between CO_2_ emissions and TACs for Cases 1 and 2. The membranes in Case 1 are D, E, and F (indicated by green diamonds), while those in Case 2 are G, H, and I (indicated by blue triangles).

In Figure 9, TAC is plotted against CO_2_ emissions for Cases 1 and 2. Note that the energy consumption, CO_2_ emissions, OPEX, and CAPEX of the membrane-separation process using Cases 1 and 2 are shown in the Supplementary Material (Figures S1–S4). The price of the membrane in Cases 1 and 2 were set higher than that of the current membranes. The reasons for this are as follows: the rise of inorganic separation membrane technology including the development of high performance supports [21] and inflation [43]. In Case 1, both the CO_2_ emissions and TACs of some membrane combinations (i.e., DE, DF, and EE) were lower than those of VRC. In Case 2, the CO_2_ emissions and TACs of most membrane combinations (i.e., GG, GH, GI, HG, HH, and HI) were lower than those of VRC. Note that TAC (0.14 MM$/y) of GG, which is the membrane combination with the lowest CO_2_ emissions (10.8 k-ton/y, 27% lower than that of VRC), was also 3% lower than that of VRC. In general, the CO_2_ emissions and TACs of most combinations of Cases 1 and 2 were lower than those of the current membrane. Note that CO_2_ emissions and TAC varies with the membrane combinations. To further investigate the membrane combinations suited for this process, the carbon price was also accounted for into TAC.

**Figure 10:**
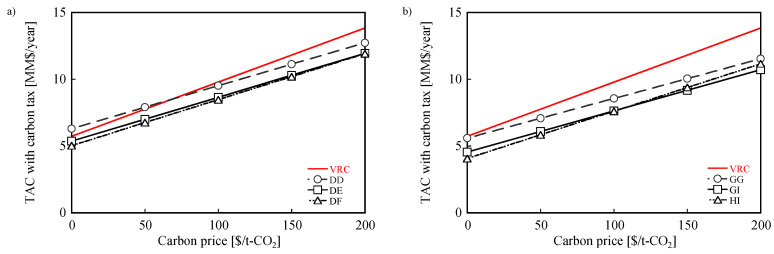
CO_2_ emissions and TACs with carbon tax for: (**a**) DD, DE, and DF in Case 1, and (**b**) GG, GI, and HI in Case 2.

Figure 10 shows TAC with the carbon tax of Cases 1 and 2 plotted against the carbon price. Three membrane combinations were selected for Cases 1 and 2: the lowest CO_2_ emissions (DD, GG); the intermediate CO_2_ emission and TAC (DE, GI); and the lowest TAC (DF, HI). In Case 1, TAC with the carbon tax of DF was the smallest within the entire range of the carbon price up to 200 $/t-CO_2_. In Case 2, TAC with the carbon tax of HI was the smallest up to the carbon price of 100 $/t-CO_2_, while that of GI was the smallest at a carbon price higher than 87 $/t-CO_2_. Thus, the best membrane combination depends on the carbon price. Note that the membrane combinations with the highest separation factors (DD and GG) are the ones with the smallest CO_2_ emission but are not the ones with the lowest TAC with carbon tax. This result indicates that it is difficult to minimize both CO_2_ emissions and TAC with carbon tax by only improving the separation performance of membranes.

## 4. Conclusions

In this study, a two-stage membrane-separation process was designed by combining membranes with different separation factors and permeance. The process performance was evaluated based on different separation factors and permeances according to the Robeson plot. Two indicators, i.e., CO_2_ emissions and TACs, were calculated using the process simulation results including energy consumptions, OPEX, and CAPEX. In this system, separation factors are the main factor for determining energy consumption, which significantly affects both OPEX and CO_2_ emissions. Membrane permeance is the main factor for determining the total area of membrane, which significantly affects CAPEX. To reduce TACs of the process, membranes with a high separation factor were used for M1, while membranes with high permeance were used for M2. Using the current membranes, which are set based on the separation performance of the zeolite, silica, and MOF membranes, the CO_2_ emissions of AA and AB were smaller than those of VRC, but TACs with the carbon tax of AA and AB were still greater than that of VRC. When the membrane performances were further improved (Cases 1 and 2), the CO_2_ emissions and TACs were lower than those of VRC. To determine the best combination for two-stage membrane systems, the CO_2_ emissions and TACs with the carbon tax were evaluated and found to depend on the carbon price.

## Figures and Tables

**Table 1:** Categories, permeance, and separation factor of the membranes used in this study.

Category	Membrane	Propylene Permeance		Propane Permeance		Separation Factor
		[mol/(m^2^ s Pa)]	[GPU] *	[mol/(m^2^ s Pa)]	[GPU] *	[–]
Current membrane	A	2.80 × 10^−8^	84	4.59 × 10^−10^	1.4	61
	B	6.10 × 10^−8^	182	1.85 × 10^−9^	5.5	33
	C	1.38 × 10^−7^	411	8.12 × 10^−9^	24	17
Case 1	D	8.10 × 10^−8^	241	8.80 × 10^−10^	2.6	92
	E	1.75 × 10^−7^	522	3.57 × 10^−9^	10	49
	F	3.90 × 10^−7^	1164	1.56 × 10^−8^	46	25
Case 2	G	2.30 × 10^−7^	686	1.69 × 10^−9^	5.0	136
	H	5.00 × 10^−7^	1492	6.85 × 10^−9^	20	73
	I	1.10 × 10^−6^	3283	2.89 × 10^−8^	86	38

* Gas permeation unit, 1 GPU = 3.35 × 10^−10^ mol/(m^2^ s Pa) [23].

**Table 2:** Feed conditions and product requirements of the membrane-separation process used in this study.

Parameters	Value
Feed temperature (K)	322
Feed pressure (kPa)	2000
Feed flow rate (kmol/h)	1589
Feed composition (propylene mole%)	90
Membrane feed-side pressure (kPa)	2000
Membrane temperature (K) [35]	373
Propylene purity (mol%)	99.5
Propylene recovery ratio (%)	99.5

**Table 3:** Feed, design, and operating conditions of CDiC.

Parameters	Value
Feed temperature (K)	322
Feed pressure (kPa)	2000
Feed flow rate (kmol/h)	1589
Feed composition (propylene mol%)	90
Feed stage ^a^	114
Total number of stages ^b^	176
Pressure at the top of the column (kPa)	2000
Reflux ratio	15.9
Condenser duty (GJ/h)	288
Reboiler duty (GJ/h)	269
Propylene purity (mol%)	99.5
Propylene recovery ratio (%)	99.5

^a^ Feed stage is numbered from top to bottom. ^b^ Number of stages includes reboiler and condenser. Number of stages was determined by the McCabe–Thiele methods.

**Table 4:** CO_2_-emission factors and utility costs.

Utility	CO_2_ Emissions [kg/GJ]	Cost [$/GJ] [43]
Steam	172	14.2
Electricity	161	16.8
Refrigerated water	54	4.4

## Data Availability

Not applicable.

## Supplementary Materials

The following supporting information can be downloaded at: https://www.mdpi.com/article/10.3390/membranes12020163/s1, Figure S1: Energy consumptions of the two-stage membrane process with various combinations. (a) Case 1 and (b) Case 2; Figure S2: CO_2_ emissions of the two-stage membrane process with various combinations. (a) Case 1 and (b) Case 2; Figure S3: The production cost of the two-stage membrane process with various combinations of Case 1: (a) OPEX and (b) CAPEX per payback period. The payback period = 4 y; Figure S4: The production cost of the two-stage membrane process with various combinations of Case 2: (a) OPEX and (b) CAPEX per payback period. The payback period = 4 y.

## Appendix A

The appendix is an optional section that can contain details and data supplemental to the main text—for example, explanations of experimental details that would disrupt the flow of the main text but nonetheless remain crucial to understanding and reproducing the research shown; figures of replicates for experiments of which representative data is shown in the main text can be added here if brief, or as Supplementary data. Mathematical proofs of results not central to the paper can be added as an appendix.

## Appendix B

**Table A1:** Design and operating conditions at minimum energy consumption for the current membranes.

Membrane		Permeate-Side Pressure [kPa]		Membrane Area [m^2^]		Energy Consumption [GJ/h]	CO_2_ Emission [k-ton/y]
M1	M2	M1	M2	M1	M2		
A	A	800	300	15,377	17,731	66.1	36.4
A	B	700	300	14,194	6860	69.4	38.0
A	C	600	300	13,180	2929	76.9	41.4
B	A	600	400	5398	33,307	86.2	45.8
B	B	600	400	5398	13,427	92.4	48.7
B	C	500	300	5038	4296	105.7	54.9
C	A	500	400	2073	43,893	124.1	63.4
C	B	400	400	1944	18,289	135.5	68.8
C	C	300	400	1829	8755	163.1	81.6
