# Supplementary material for: Design and Evaluation of Two-Stage Membrane-Separation Processes for Propylene–Propane Mixtures
Source: Membranes (Basel). 2022 Jan 29;12(2):163. doi: 10.3390/membranes12020163 (PMC8874774; doi:10.3390/membranes12020163)
Supplement: Supplementary file 1 [file membranes-12-00163-s001.zip › membranes-1550372-supplementary.pdf]

membranes

MDPI

Article

Design and Evaluation of Two-Stage Membrane-Separation Processes for Propylene–Propane Mixtures

Takehiro Yamaki *, Nguyen Thuy, Nobuo Hara, Satoshi Taniguchi and Sho Kataoka *

Research Institute for Chemical Process Technology, National Institute of Advanced Industrial Science and Technology (AIST), AIST Tsukuba Central 5, 1-1-1 Higashi, Tsukuba 305-8565, Ibaraki, Japan; nguyen.thuy@aist.go.jp (N.T.); n-hara@aist.go.jp (N.H.); taniguchi-satoshi@aist.go.jp (S.T.)

* Correspondence: takehiro-yamaki@aist.go.jp (T.Y.); s-kataoka@aist.go.jp (S.K.)

Citation: Yamaki, T.; Thuy, N.; Hara, N.; Taniguchi, S.; Kataoka, S. Design and Evaluation of Two-Stage Membrane-Separation Processes for Propylene-Propane Mixtures. *Membranes* **2022**, *12*, 163. https://doi.org/10.3390/membranes12020163

Academic Editor: Tomohisa Yoshioka

Received: 27 December 2021
Accepted: 25 January 2022
Published: 29 January 2022

Publisher's Note: MDPI stays neutral with regard to jurisdictional claims in published maps and institutional affiliations.

Copyright: © 2022 by the authors. Submitted for possible open access publication under the terms and conditions of the Creative Commons Attribution (CC BY) license (https://creativecommons.org/licenses/by/4.0/).

Supplementary data

Figures S1 and S2 show the energy consumption and CO_2 emissions, respectively, of the membrane-separation process in Cases 1 and 2.

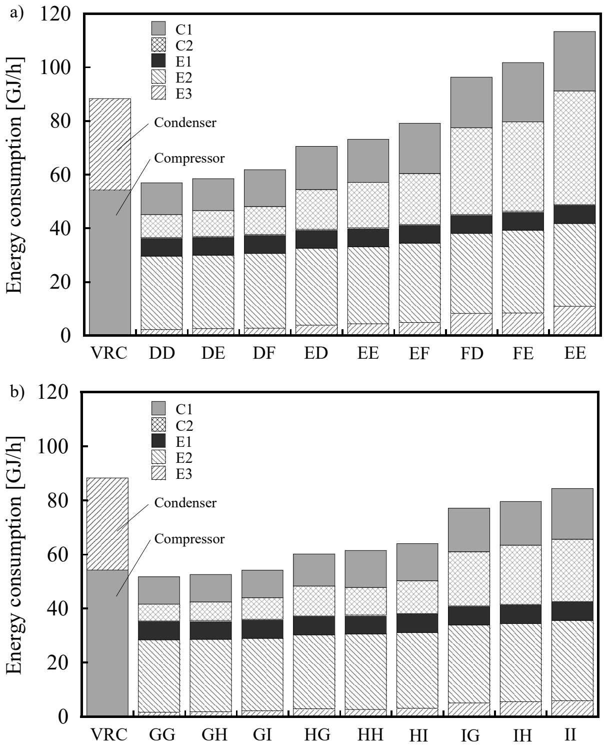

Figure S1. Energy consumptions of the two-stage membrane process with various combinations. (a) Case 1 and (b) Case 2.

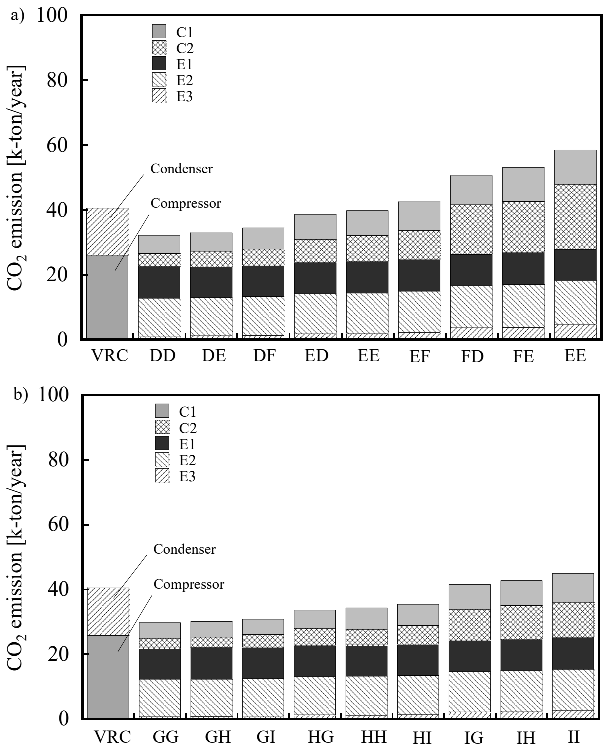

Figure S2. CO_2 emissions of the two-stage membrane process with various combinations. a) Case 1 and b) Case 2.

Figures S3 and S4 show the OPEX and CAPEX of the membrane-separation process for Cases 1 and 2, respectively.

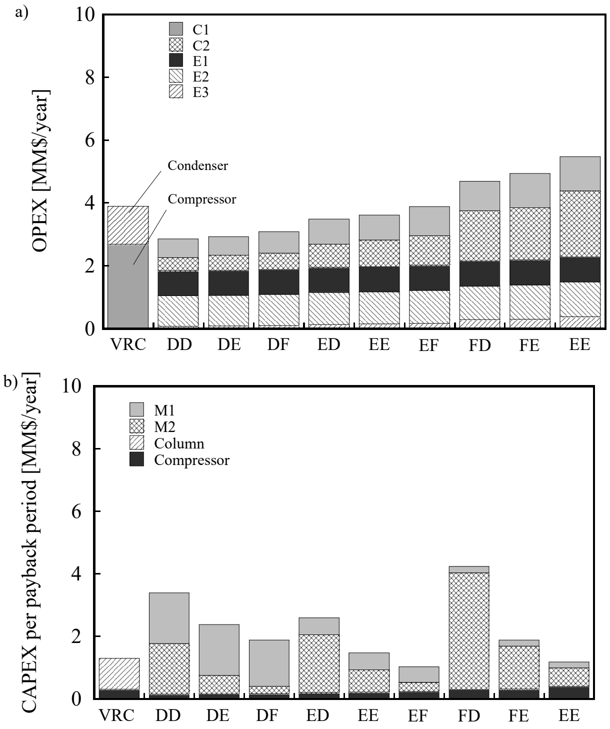

Figure S3. The production cost of the two-stage membrane process with various combinations of Case 1: a) OPEX and b) CAPEX per payback period. The payback period = 4 y.

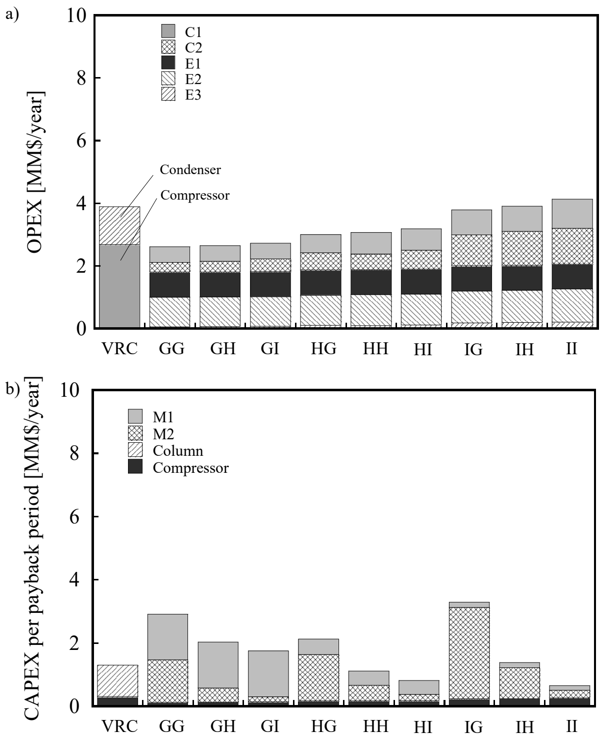

Figure S4. The production cost of the two-stage membrane process with various combinations of Case 2: a) OPEX and b) CAPEX per payback period. The payback period = 4 y.
